# A gene-based pangenome reveals genomic divergence and altitudinal differentiation in allotetraploid quinoa

**DOI:** 10.3389/fpls.2026.1930221

**Published:** 2026-09-07

**Authors:** Yanyan Pu, Liwen Wang, Yongchao Gong, Jingyin Yu, Nana Li, Xingli Dai, Runfang Li, Miaomiao Huang, Jikai Zhang, Peng Zheng, Wei Jiang, Hongjun Zhao

**Affiliations:** 1 Shandong Academy of Agricultural Sciences, Jinan, China; 2 School of Life Sciences, Qilu Normal University, Jinan, China; 3 Xianghu Laboratory, Hangzhou, China; 4 Shandong Seed Industry Intelligent Science Agriculture Service Group Co., Ltd, Jinan, China

**Keywords:** breeding, gene-based pangenome, genetic diversity, PAV, positive selection, quinoa

## Abstract

Quinoa (*Chenopodium quinoa* Willd.) is an allotetraploid crop with extraordinary environmental adaptability. To explore its genetic diversity, evolutionary dynamics, and altitudinal differentiation, we constructed a gene-based pangenome using 11 genomes (7 highland and 4 lowland accessions), comprising 51,298 orthologous groups with 19,653 core pan-genes, suggesting a near-closed pangenome architecture within the sampled accessions. Evolutionary analysis revealed that core pan-genes experienced the strongest purifying selection, whereas private pan-genes exhibited the weakest purifying selection, though the difference was not statistically significant compared to the other three pan-gene sets. Subgenomic asymmetry analysis showed that subgenome A retained fewer single-copy genes than subgenome B, with dosage-sensitive two-copy genes enriched in biotic stress-related domains and tandemly duplicated genes associated with abiotic stress adaptation. Presence/absence variation (PAV) analysis identified 7,477 orthologous groups enriched in highland accessions and 3,549 enriched in lowland accessions. We caution that these counts may be substantially inflated by the uneven sample size (7 highland vs. 4 lowland accessions) and should be interpreted as descriptive enrichment patterns rather than definitive evidence of ecotype divergence. Highland-enriched genes were enriched in transposon-related families and FAR1 domains potentially enhancing genomic plasticity and light signaling, whereas lowland-enriched genes were enriched in nitrogen metabolism and defense-related families. Copy number variation of betalain biosynthetic genes (CqCYP76AD, CqDODA, CqDOPA5-GT) was not the primary driver of seed coat color divergence between highland and lowland ecotypes. Four positively selected genes, including NADH-quinone oxidoreductase and MT-A70, harbor population-specific amino acid substitutions that may contribute to differential adaptation, potentially optimizing photosynthetic metabolism, RNA epigenetic modification, and protein homeostasis. These findings provide insights into the genomic basis of quinoa’s environmental adaptation and altitudinal differentiation, offering a genomic resource for molecular breeding.

## Introduction

Quinoa (*Chenopodium quinoa* Willd., 2n = 4x = 36, AABB), one of the most important pseudocereals cultivated worldwide, belongs to the genus *Chenopodium* in the Amaranthaceae family (Jarvis et al., 2017). It has been praised as “the mother grain” due to its comprehensive nutritional profile and exceptional stress tolerance, attracting increasing global attention (Gordillo-Bastidas et al., 2016; Yasui et al., 2016). Quinoa is an allotetraploid species that originated from the hybridization of two distinct diploid ancestors approximately 3.3–6.3 million years ago during the late Miocene to early Pliocene (Yasui et al., 2016). The A subgenome was likely donated by an ancestor related to the extant diploid *C. watsonii* (2n = 2x = 18), a species native to the Sonoran Desert of the southwestern United States with high sequence similarity to the quinoa A subgenome (>99.3% sequence identity), while the B subgenome was contributed by an ancestor related to the North American coastal diploid *C. suecicum* or other species in the *C. ficifolium/C. berlandieri* complex (Kolano et al., 2016; Rey et al., 2023; Young et al., 2023). This allopolyploid origin has endowed quinoa with remarkable genetic diversity and adaptive plasticity, facilitating its colonization of diverse agro-ecological zones from sea level to 4,000 meters elevation in the Andes (Rey et al., 2024).

Among modern cultivated quinoa, there are distinct ecotypes, including the Andean highland type and the Chilean coastal lowland type, indicative of breeding goals that have targeted adaptation to diverse production environments (Rey et al., 2023; Zurita-Silva et al., 2014). Notably, the evolution and domestication of quinoa species have been accompanied by changes in gene dosage and function that gave rise to variation in important traits or phenotypes among various quinoa accessions, such as flowering time, grain size, protein content, and stress resistance (Jaggi et al., 2025; Zurita-Silva et al., 2014).

Over the past decade, several genome assemblies have been generated for quinoa (2n = 4x = 36), playing a pivotal role in advancing our understanding of its genetic diversity and laying a solid foundation for subsequent genetic studies. Draft assemblies have been produced for four distinct accessions: Kd, a Japanese inbred line (Yasui et al., 2016); PI 614886 (QQ74), a coastal Chilean lowland ecotype (Jarvis et al., 2017); Real, a highland cultivar representing one of the most widely grown commercial varieties (Zou et al., 2017); and CHEN125, a Bolivian landrace (Bodrug et al., 2021). More recently, an improved version of the coastal quinoa assembly (QQ74-V2) was generated with substantially enhanced contiguity and completeness, thereby enabling larger-scale and more precise genetic investigations (Rey et al., 2023). Additionally, a panel of eight accessions was strategically selected to capture the broad phenotypic diversity of quinoa, comprising three lowland genotypes: Regalona and Javi from Chile, and D-12282 from Argentina, and five highland genotypes: CHEN-199 and PI-614919 from Bolivia, and 03-21-072RM, D-10126, and CHEN-90 from Peru (Rey et al., 2024). Although a reference crop genome is a good starting point to assess the basis of complex agronomic traits, a gene-based pangenome provides insights into the genetic diversity of a crop and its relatives.

To explore the genetic diversity, evolutionary dynamics, and adaptive mechanisms of quinoa, we constructed a gene-based pangenome using 11 accessions (7 highland and 4 lowland), comprising 504,240 representative genes classified into core, softcore, dispensable, and private sets. Our analyses revealed a closed pangenome architecture with asymmetric subgenome evolution, where core genes experienced strong purifying selection while non-core genes enriched in stress-related functions supported adaptive evolution. Phylogenetic and PAV analyses further uncovered distinct evolutionary trajectories between highland and lowland ecotypes, identifying candidate genes underlying their adaptive divergence. These findings provide a comprehensive genomic resource and lay a solid foundation for quinoa molecular breeding.

## Results

### High-quality quinoa gene-based pangenome

**Figure 1:**
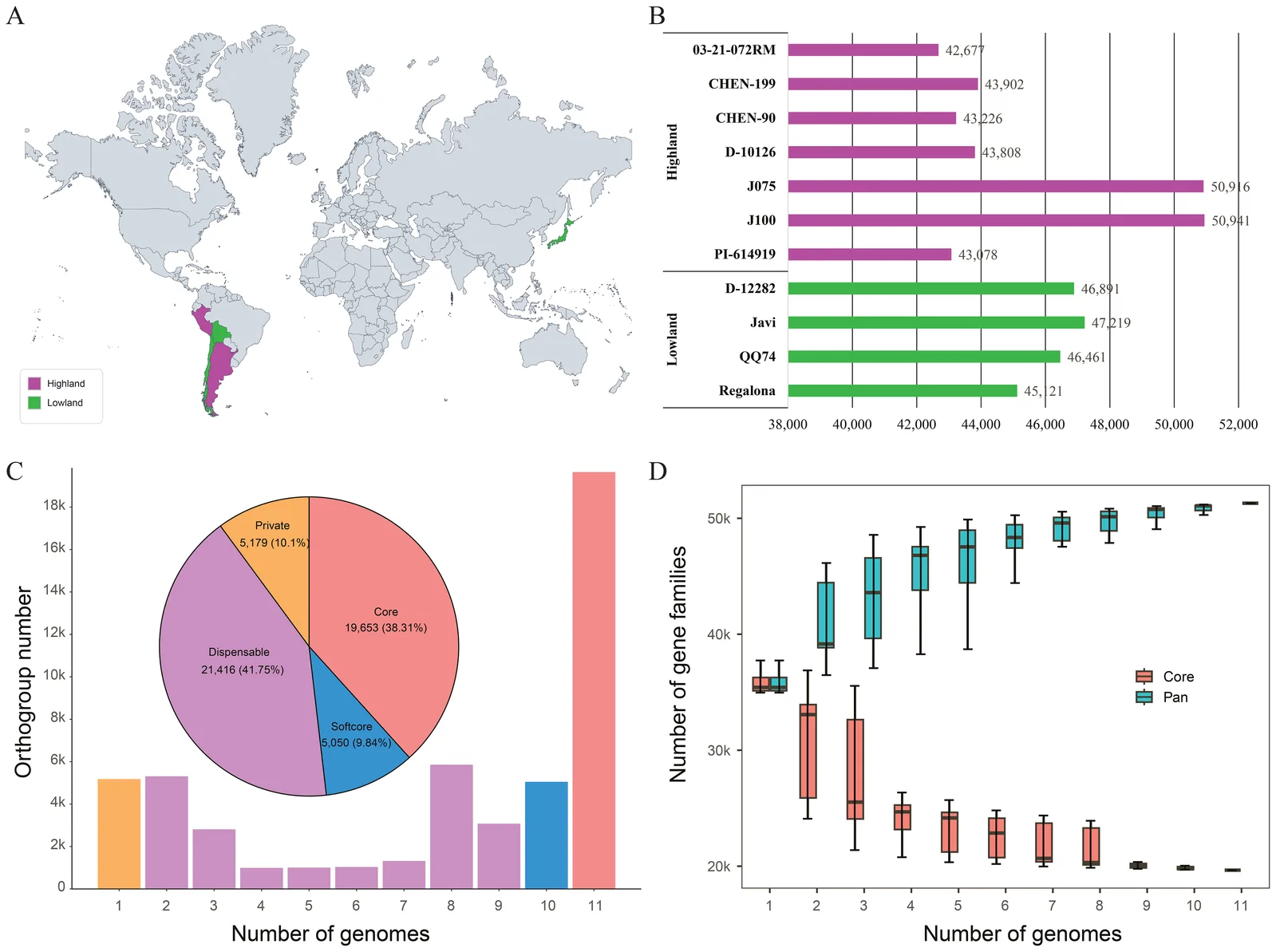
Statistics and phylogeny of 11 quinoa genomes. **(A)** Geographical distribution of 11 quinoa accessions; **(B)** the number of representative genes in each quinoa genome; **(C)** Composition of the quinoa gene-based pangenome; **(D)** Simulations of the pangenome and core-genome size in the pangenome.

A total of 11 quinoa genomes, comprising seven highland and four lowland accessions, were used to construct a gene-based pangenome (**Figure 1A**; **Supplementary Table 1**). For each genome, the longest transcript per locus was designated as the representative gene. Transcripts containing transposable elements were subsequently removed from the gene sets. The final representative gene set ranged from 42,677 (*C. quinoa* acc. 03-21-072RM) to 50,941 (*C. quinoa* acc. J100) (**Figure 1B**). BUSCO assessment using the embryophyta_odb10 lineage database indicated high completeness of gene annotations, ranging from 94.5% (*C. quinoa* acc. 03-21-072RM) to 98.6% (*C. quinoa* acc. QQ74) (Sima et al., 2015). Totally, 504,240 representative genes were retained for pangenome construction (**Table 1**).

**Table 1:** Statistics of quinoa genomes.

Species	Accession name	Origin	Ecotype	Total genes	Total gene model	Representative gene model	BUSCO evaluation	Reference
*C. quinoa*	03-21-072RM	Peru	Highland	43,733	69,168	42,677	94.50%	Rey et al., 2024
*C. quinoa*	CHEN-199	Bolivia	Highland	45,013	94,700	43,902	95.60%	Rey et al., 2024
*C. quinoa*	CHEN-90	Peru	Highland	44,345	92,962	43,226	95.40%	Rey et al., 2024
*C. quinoa*	D-10126	Peru	Highland	44,920	94,607	43,808	95.50%	Rey et al., 2024
*C. quinoa*	D-12282	Argentina	Lowland	48,201	99,014	46,891	95.80%	Rey et al., 2024
*C. quinoa*	J075	Peru	Highland	57,061	65,303	50,916	94.80%	Kobayashi et al., 2024
*C. quinoa*	J100	Bolivia	Highland	56,657	64,945	50,941	94.80%	Kobayashi et al., 2024
*C. quinoa*	Javi	Chile	Lowland	48,564	99,431	47,219	95.70%	Rey et al., 2024
*C. quinoa*	PI-614919	Bolivia	Highland	44,183	92,859	43,078	95.30%	Rey et al., 2024
*C. quinoa*	QQ74	Chile	Lowland	48,375	54,339	46,461	98.60%	Rey et al., 2023
*C. quinoa*	Regalona	Chile	Lowland	46,302	68,963	45,121	94.70%	Rey et al., 2024

The resulting pangenome comprised 51,298 orthologous groups, of which 19,653 (38.31% of total groups) were present in all 11 quinoa genomes and classified as the core pan-gene set. Additionally, 5,050 (9.84%), 21,416 (41.75%), and 5,179 (10.1%) groups were categorized as softcore (present in 10–11 genomes, ≥90.9%), dispensable (present in 2–9genomes), and private (present in only one genome) pan-gene sets, respectively (**Figure 1C**). The private pan-gene set consisted of 5,179 singleton orthologous groups and 639 multi-copy orthologous groups specific to individual accessions. Modeling of the quinoa gene-based pangenome indicated the gene repertoire is approaching saturation (near-closed architecture) within the 11-accession panel, with 95% confidence intervals derived from bootstrap resampling (**Figure 1D**).

### Functional divergence of pan-gene sets

**Figure 2:**
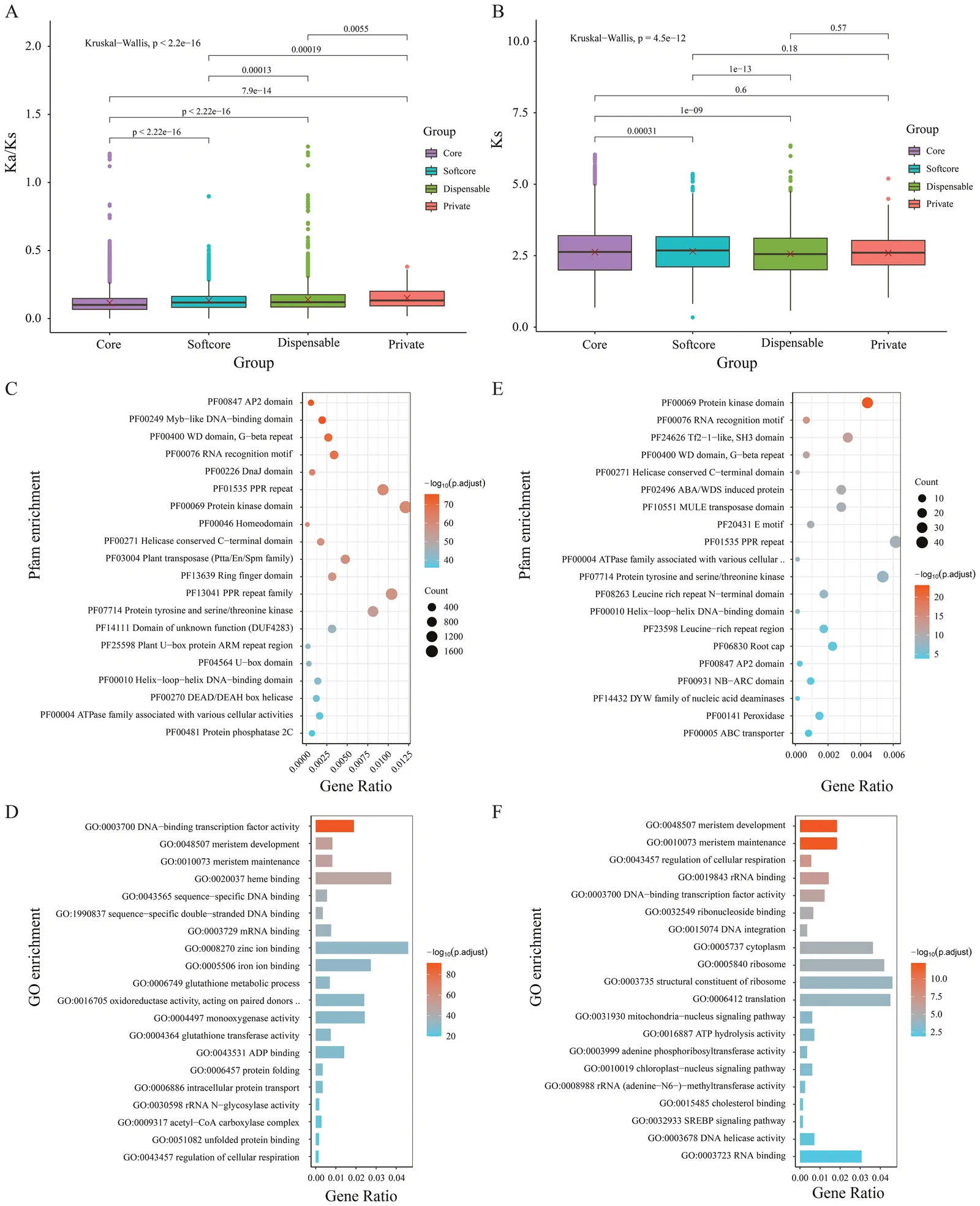
Evolution and divergence of the dispensable and private pan-genes in the quinoa gene-based pangenome. **(A)** *K*a/*K*s analyses statistics of core, softcore, dispensable, and private pan-genes; **(B)** *K*s analyses statistics of core, softcore, dispensable, and private pan-genes; **(C, D)** Pfam and GO (gene ontology) enrichment analysis of the dispensable pan-genes; **(E, F)** Pfam and GO enrichment analysis of the private pan-genes.

The Ka/Ks values of orthologous gene pairs between grape and quinoa were calculated to investigate functional divergence among core, softcore, dispensable, and private pan-gene sets in the quinoa pangenome. All Ka/Ks values were significantly less than 1 (Fisher’s exact test, P < 0.05), indicating that all four pan-gene categories have been subject to purifying (negative) selection. The mean Ka/Ks value was lowest for core pan-genes (0.1172) and highest for private pan-genes (0.1574) (Fisher’s exact test, P < 0.05), suggesting that core pan-genes have experienced the strongest purifying selection pressure and are thus the most evolutionarily constrained. However, statistical analysis revealed no significant differences in Ka/Ks values between private pan-genes and the other three pan-gene categories (**Figure 2A**). Regarding synonymous substitution rates (Ks), the mean Ks value was highest for private pan-genes (2.525) and lowest for softcore pan-genes (0.7637) (Fisher’s exact test, P < 0.05), indicating that private pan-genes have undergone more sequence divergence. Nevertheless, no significant differences in Ks values were detected between private pan-genes and the other three categories (**Figure 2B**). Pfam enrichment analysis revealed that dispensable pan-genes were significantly enriched in protein kinases and transcription factors (**Figure 2C**; **Supplementary Table 2**), which are primarily involved in binding and catalytic activities (**Figure 2D**; **Supplementary Table 3**). Private pan-genes were enriched in protein kinases, transcription factors, and disease resistance genes, with NBS-LRR genes being the most abundant disease resistance gene family (**Figure 2E**; **Supplementary Table 4**). Interestingly, many private pan-genes were enriched in the same gene families as those in the dispensable set, suggesting that these gene superfamilies have undergone strong diversification to generate genome-specific members across different quinoa accessions. These genes are mainly involved in meristem development and maintenance (**Figure 2F**; **Supplementary Table 5**).

### New phylogeny of highland and lowland quinoa

**Figure 3:**
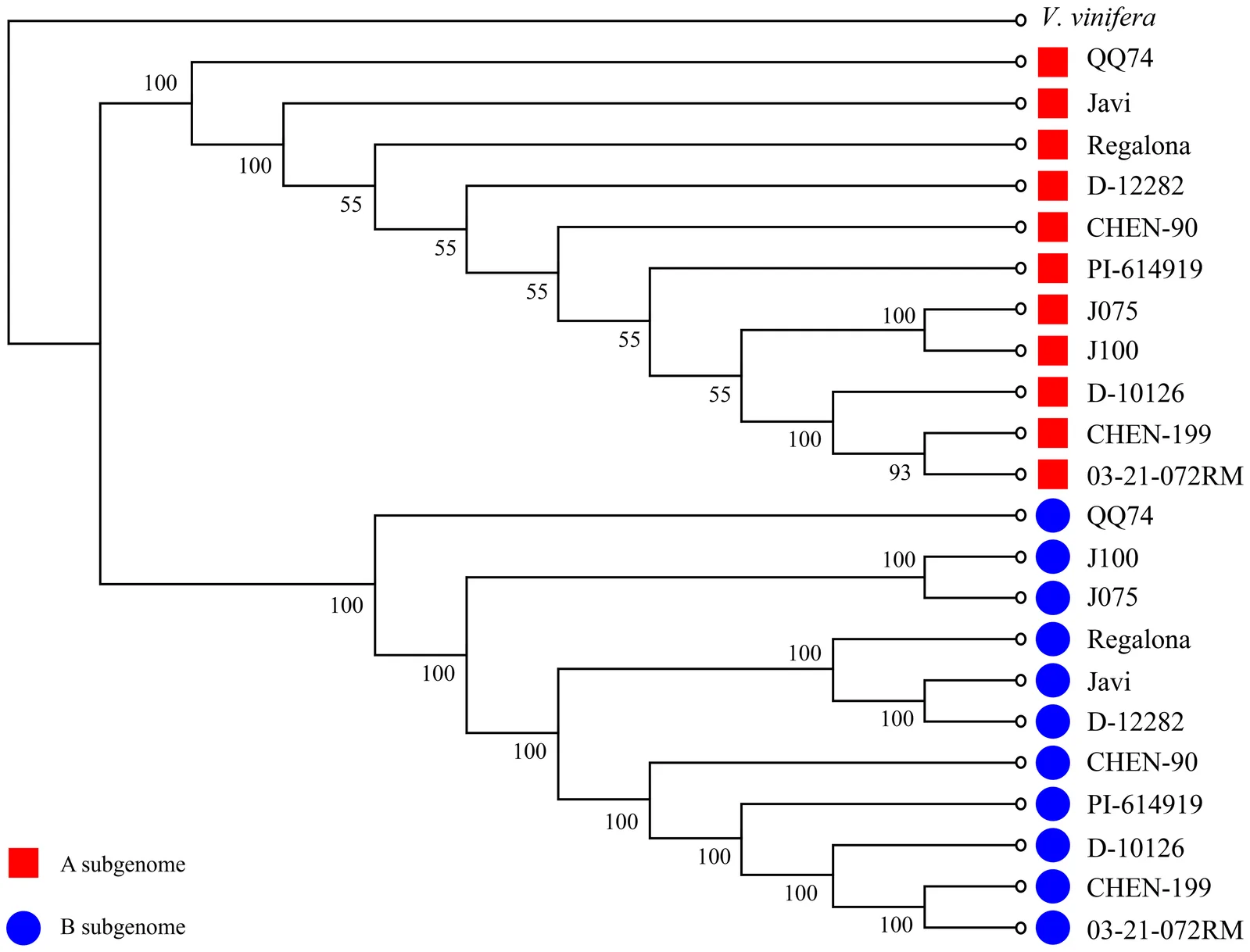
Phylogeny analysis of A and B subgenomes of 11 quinoa accessions. Filled red squares denote the A subgenome, and filled blue circles denote the B subgenome, across 11 quinoa accessions. Phylogenetic branches bearing red solid squares are assigned to the “Subgenome A” clade, whereas those bearing blue solid circles are assigned to the “Subgenome B” clade.

Quinoa is an allotetraploid species that originated from hybridization between a North American diploid progenitor (A subgenome donor) and a Eurasian diploid progenitor (B subgenome donor) (Young et al., 2023). To investigate the evolutionary history of various quinoa varieties, we partitioned 11 allotetraploid genomes into 22 A and B subgenomes. Using a true diploid grape (*Vitis vinifera*) as the outgroup, a maximum-likelihood phylogenetic tree of the 22 subgenomes resolved into two major clades: “Subgenome A” and “Subgenome B” (**Figure 3**). All 11 A subgenomes clustered within the Subgenome A clade, whereas all 11 B subgenomes clustered within the Subgenome B clade. Phylogenetic analysis revealed that both A and B subgenomes of the coastal Chilean accession, *C. quinoa* acc. QQ74, exhibited shorter branch lengths to the outgroup grape than those of other quinoa accessions, indicating a shorter genetic distance to the outgroup and potentially a lower apparent substitution rate. Conversely, three highland quinoa accessions (*C. quinoa* acc. D-10126 and *C. quinoa* acc. 03-21-072RM from Peru; *C. quinoa* acc. CHEN-199 from Bolivia) showed both subgenomes to have longer branch lengths to grape than other accessions, suggestive of greater sequence divergence or higher substitution rates. Within the Subgenome A clade, excluding *C. quinoa* acc. QQ74, the A subgenomes of three lowland accessions (*C. quinoa* acc. Javi, *C. quinoa* acc. Regalona, and *C. quinoa* acc. D-12282) were positioned closer to the grape outgroup than all highland accessions. Within the Subgenome B clade, the B subgenomes of two highland accessions (*C. quinoa* acc. J075 and *C. quinoa* acc. J100) were more closely related to *C. quinoa* acc. QQ74 than those of the three lowland accessions, which formed a distinct group with shorter branch lengths than those of highland accessions (*C. quinoa* acc. D-10126, *C. quinoa* acc. 03-21-072RM, and *C. quinoa* acc. CHEN-199). We caution that differences in branch length among extant accessions do not reflect absolute evolutionary age, as all accessions have evolved for the same amount of time since their common ancestor. Rather, these patterns likely reflect variation in substitution rates, demographic histories, or technical factors such as alignment quality and annotation completeness. Within the Subgenome A clade, excluding *C. quinoa* acc. QQ74, the A subgenomes of three lowland accessions (*C. quinoa* acc. Javi, *C. quinoa* acc. Regalona, and *C. quinoa* acc. D-12282) were positioned closer to the quinoa progenitor than all highland accessions. Within the Subgenome B clade, the B subgenomes of two highland accessions (*C. quinoa* acc. J075 and *C. quinoa* acc. J100) were more closely related to *C. quinoa* acc. QQ74 than those of the three lowland accessions, which formed a distinct group and appeared more ancestral than those of highland accessions (*C. quinoa* acc. D-10126, *C. quinoa* acc. 03-21-072RM, and *C. quinoa* acc. CHEN-199). Collectively, these results indicate that out of lowland accessions, *C. quinoa* acc. QQ74 conserves the most ancient evolutionary legacy, while the highland accession *C. quinoa* acc. 03-21-072RM possesses the most recently evolved traces among the 11 quinoa accessions.

We note that the phylogenetic branch length patterns observed here should not be interpreted as evidence of absolute evolutionary age. All extant quinoa accessions have evolved for the same amount of time since their common allotetraploid ancestor; therefore, longer branches do not indicate “more recent evolution” and shorter branches do not indicate “more ancient” status. Instead, the observed variation in branch length to the grape outgroup likely reflects differences in substitution rates, effective population sizes, or demographic histories among accessions. The shorter branches observed in C. quinoa acc. QQ74 may reflect a lower substitution rate or larger effective population size in the coastal lowland lineage, whereas the longer branches in some highland accessions may reflect higher substitution rates or greater drift. Alternatively, technical factors such as differences in assembly quality, annotation completeness, or alignment accuracy may contribute to apparent branch length variation. We have removed all previous interpretations linking branch length to “ancient” or “recently evolved” status.

### Asymmetric evolution of subgenomes in quinoa genome

**Figure 4:**
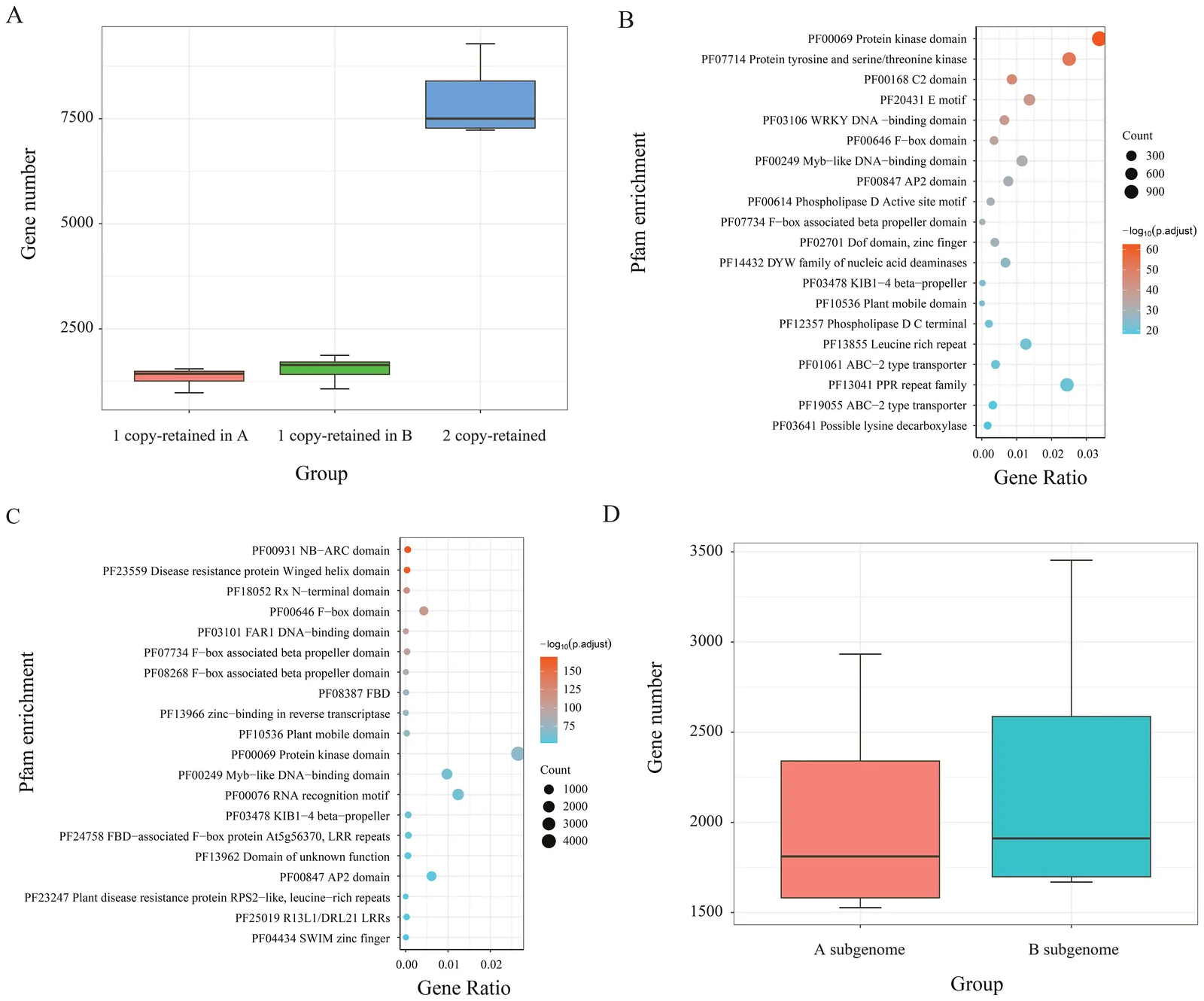
Gene retention on quinoa A and B subgenomes. **(A)** Comparison of single-copy and two-copy retained genes on A and B subgenomes; **(B)** Pfam enrichment analysis of single-copy retained genes in A or B subgenomes; **(C)** Pfam enrichment analysis of two-copy retained genes in A and B subgenomes; **(D)** Comparison of tandemly duplicated genes on A and B subgenomes.

Genome analyses revealed that the closest extant relatives of quinoa A and B subgenomes, *C. watsonii* and *C. ficifolium*, respectively (Ludwig et al., 2025; Young et al., 2023), along with *V. vinifera*, have not undergone any whole-genome duplication (WGD) events after the angiosperm-wide γ triplication (Jaillon et al., 2007). *V. vinifera* was used as the outgroup to identify single-copy and two-copy orthologs in both quinoa subgenomes. Orthologous relationships between *V. vinifera* and individual quinoa subgenomes were established to characterize biased evolutionary patterns among subgenomes. Based on subgenomic distribution, orthologs were categorized into two groups: single-copy retained genes (present exclusively in either subgenome A or B) and two-copy retained genes (retained in both subgenomes). Subgenomic retention analysis indicated that subgenome A retained fewer single-copy genes than subgenome B in each quinoa accession, whereas two-copy retained genes were approximately twice as abundant as all single-copy retained genes combined across the 11 quinoa genomes (**Figure 4A**; **Supplementary Table 6**). Collectively, these findings reveal evolutionary asymmetry between quinoa subgenomes, manifested as biased retention of single-copy genes relative to *V. vinifera*. This is consistent with previous reports that polyploidization often leads to asymmetric subgenome evolution, wherein one subgenome dominates in gene retention. Single-copy and two-copy retained genes in allotetraploid genomes are classified as subgenome-dominant and dosage-sensitive genes, respectively. Two-copy retained genes are subject to neofunctionalization, subfunctionalization, or functional decay following gene dosage increase. Protein family enrichment analysis revealed that single-copy retained genes were mainly enriched in protein kinase domain, protein tyrosine and serine/threonine kinase, C2 domain, E motif, WRKY DNA −binding domain, F−box domain, Myb−like DNA−binding domain, AP2 domain, Phospholipase D Active site motif, and F−box associated beta propeller domain (Bonferroni-adjusted *P* value < 0.05) (**Figure 4B**; **Supplementary Table 7**), whereas two-copy retained genes were predominantly enriched in NB−ARC domain, Disease resistance protein Winged helix domain, Rx N−terminal domain, F−box domain, FAR1 DNA−binding domain, F−box associated beta propeller domain, FBD, zinc−binding in reverse transcriptase, and plant mobile domain (Bonferroni-adjusted *P* value < 0.05) (**Figure 4C**; **Supplementary Table 8**). So, the dosage-sensitive genes in quinoa genomes may facilitate disease resistance. Furthermore, tandemly duplicated genes were detected across the quinoa accessions, with copy numbers ranging from 3,196 (*C. quinoa* acc. CHEN-90) to 6,387 (*C. quinoa* acc. QQ74). These duplicates constituted 7.39% to 13.75% of the total protein-coding genes in the corresponding genomes. A total of 46,004 tandemly duplicated genes were identified in the quinoa gene-based pangenome, distributed across tandem arrays ranging from 2 to 26 genes in size. Tandem duplicated genes were less abundant in subgenome A than in subgenome B across all quinoa genomes indicating the subgenome asymmetry of tandem duplication events (**Figure 4D**; **Supplementary Table 9**). Most of TD genes were found to be enriched in the gene families of F-box domain, UDP-glucoronosyl and UDP-glucosyl transferase, Cupin, Cytochrome P450, Salt stress response/antifungal, 2OG-Fe(II) oxygenase superfamily, non-haem dioxygenase in morphine synthesis N-terminal, Protein BPS1, Glutathione S-transferase, N-terminal domain, and Peroxidase (Bonferroni-adjusted *P* value < 0.05) (**Supplementary Figure 2**; **Supplementary Table 10**). Interestingly, the enriched protein families of dosage-sensitive genes derived from quinoa polyploidization events are primarily involved in biotic stress tolerance, whereas those of tandemly duplicated genes originating from tandem duplication events are mainly associated with abiotic stress tolerance. These two distinct evolutionary mechanisms both contribute to increased gene dosage, thereby enhancing environmental adaptation in quinoa accessions.

### Presence/absence variation between highland and lowland quinoas

**Figure 5:**
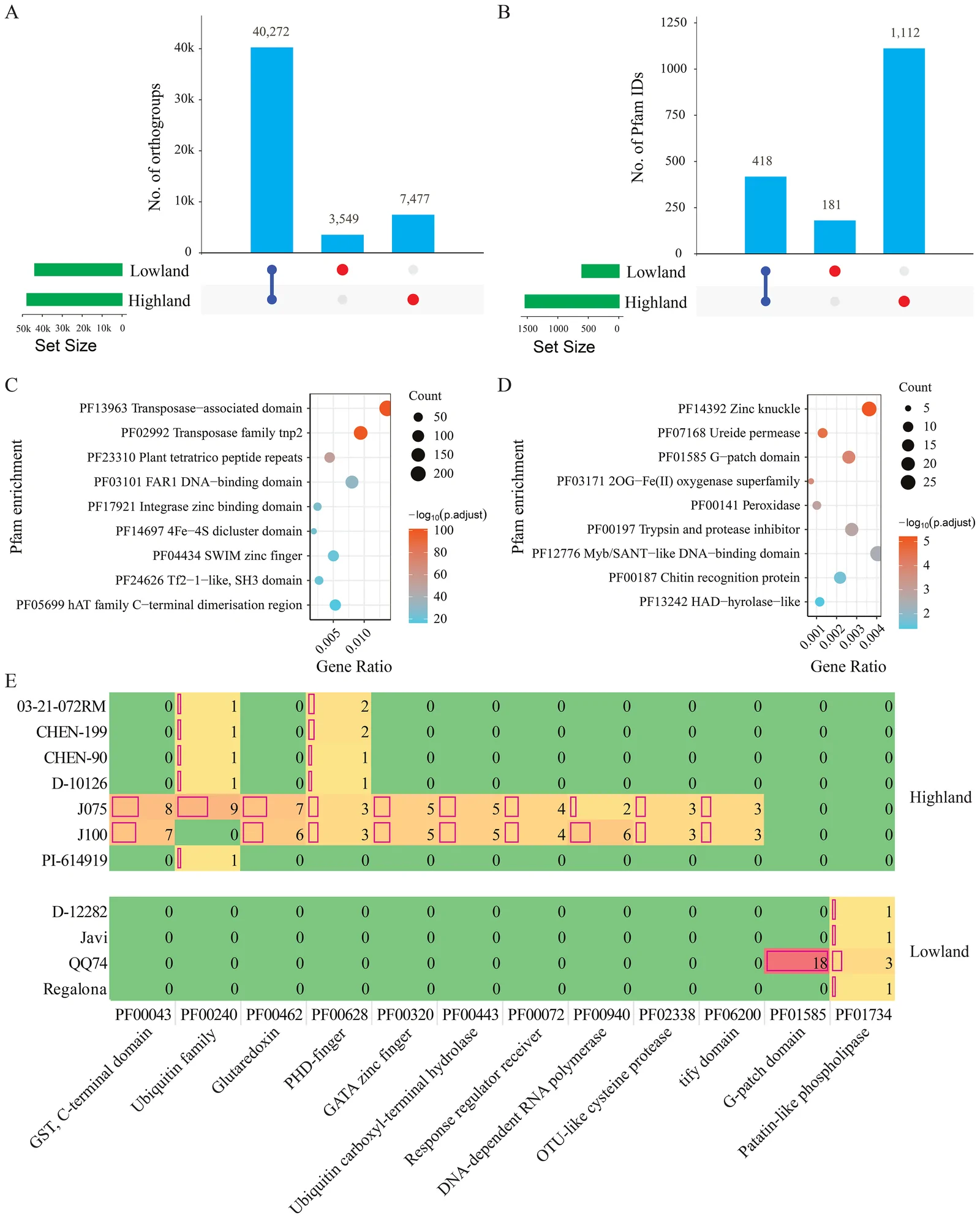
Genes or gene families that have been affected during evolution and domestication in the quinoa clade. **(A)** Variation of orthologous groups in the two quinoa taxa. Red solid circles represent specific orthologous groups; **(B)** Variation of Pfam IDs in the two quinoa taxa. Red solid circles represent specific Pfam IDs; **(C)** Pfam enrichment analysis of specific genes in highland quinoa accessions; **(D)** Pfam enrichment analysis of specific genes in lowland quinoa accessions; **(E)** Genes related to growth and development, and stress responses in quinoa individuals. Yellow rectangles represent the presence of target genes and red border rectangles represent the numbers of target genes in quinoa individuals.

Presence/absence variation (PAV) refers to a type of structural genomic variation in which a DNA sequence (typically containing one or more genes) is present in some individuals but entirely absent in others within the same species (Bush et al., 2013). Such variation drives genetic divergence and phenotypic diversity among different accessions, thereby enhancing environmental adaptability. It represents an important evolutionary strategy that enables plants to survive and thrive in extreme environments. The highland and lowland quinoa accessions provide an opportunity to investigate presence/absence variable genes under different growth conditions (Mizuno et al., 2020). Among 51,298 orthologous groups identified from the quinoa gene-based pangenome, 7,477 and 3,549 accession-specific orthologous groups containing 16,333 and 6,899 protein-coding genes were detected in highland and lowland quinoa, respectively (**Figure 5A**). We emphasize that the unequal sample size (7 highland vs. 4 lowland accessions) likely inflates the count of genes enriched in highland accessions. Consequently, these figures represent descriptive enrichment patterns within the current, unbalanced panel rather than exclusive, ecotype-defining gene sets. A balanced sampling design is required to rigorously assess PAV differences between altitudinal ecotypes. Most accession-specific genes were annotated with protein families. Among the annotated Pfam IDs, 1,112 were specific to highland quinoa accessions, whereas 181 were specific to lowland quinoa accessions (**Figure 5B**). The specific genes in highland quinoa accessions mainly enriched in the families of transposase-associated, transposase family tnp2, plant tetratrico peptide repeats, FAR1 DNA-binding, integrase zinc binding, 4Fe-4S dicluster, SWIM zinc finger, Tf2-1-like, SH3, and hAT family C-terminal dimerisation region (Bonferroni-adjusted *P* value < 0.05) (**Figure 5C**; **Supplementary Table 11**). This analysis reveals that high-altitude quinoa enhances genomic plasticity through transposon-related gene families (PF13963, PF02992, PF05699) and optimizes light signaling perception via FAR1 domains (PF03101) to adapt to extreme environments, while 4Fe-4S dicluster domains (PF14697) function as redox sensors coordinating with TPR, SWIM, and SH3 domain protein networks to maintain growth homeostasis under stress, collectively enabling successful adaptation to the Andean plateau. The specific genes in lowland quinoa accessions only enriched in the families of Zinc knuckle, Ureide permease, G-patch, 2OG-Fe(II) oxygenase superfamily, Peroxidase, Trypsin and protease inhibitor, Myb/SANT-like DNA-binding, Chitin recognition protein, and HAD-hyrolase-like (Bonferroni-adjusted *P* value < 0.05) (**Figure 5D**; **Supplementary Table 12**). This analysis reveals that lowland quinoa prioritizes metabolic efficiency and biotic defense over genomic plasticity, as evidenced by the enrichment of ureide permease and HAD-hydrolase for nitrogen assimilation, alongside chitin recognition proteins and protease inhibitors for pathogen and herbivore resistance; concurrently, the expansion of Myb/SANT transcription factors and 2OG-Fe(II) oxygenase superfamily members enables fine-tuned developmental regulation and secondary metabolism, collectively forming a “defense-efficient, metabolically optimized” strategy distinct from the transposon-driven adaptation of high-altitude accessions.

Moreover, we conducted a comparative presence/absence variation (PAV) analysis of disease resistance genes to investigate environmentally adaptive differences between highland and lowland quinoa accessions. We detected 15 plant–pathogen interaction genes containing the glutathione S-transferase C-terminal domain in two highland quinoa accessions, *C. quinoa* acc. J075 and J100 (**Figure 5E**). Nine members of the ubiquitin family, which are involved in plant interactions with pathogens and other microbes, were identified exclusively in the highland quinoa accession *C. quinoa* acc. J100. Among the seven highland quinoa accessions analyzed, only one ubiquitin family member was present in five accessions: *C. quinoa* acc. 03-21-072RM, CHEN-199, CHEN-90, D-10126, and PI-614919. PHD finger proteins participate in plant development and abiotic stress responses. A total of 12 PHD finger genes were identified in highland quinoa accessions, distributed as follows: two in *C. quinoa* acc. 03-21-072RM, two in acc. CHEN-199, one in acc. CHEN-90, one in acc. D-10126, three in acc. J075, and three in acc. J100. The G-patch domain is widely conserved among eukaryotic proteins, spanning approximately 48 amino acid residues. Its most distinctive feature is the presence of six highly conserved amino acid residues. For example, MOS2—a protein containing both G-patch and KOW motifs—is essential for innate immunity in *Arabidopsis*. In this study, we identified 18 protein-coding genes containing the G-patch domain that were exclusively present in the lowland quinoa accession *C. quinoa* acc. QQ74. Patatin-like phospholipases are involved in plant cell death and defense signaling; silencing of *CaPLP1* in pepper conferred enhanced susceptibility to avirulent *Xanthomonas campestris* pv. *vesicatoria* infection. In this analysis, four genes encoding patatin-like phospholipase were identified in two lowland quinoa accessions: *C. quinoa* acc. QQ74 (3) and Regalona (1). Notably, the uneven sample size (7 highland/4 lowland accessions) may partially inflate the count of highland-enriched orthogroups; permutation resampling could further normalize this bias in follow-up work.

### Quinoa seed coat color related genes

**Figure 6:**
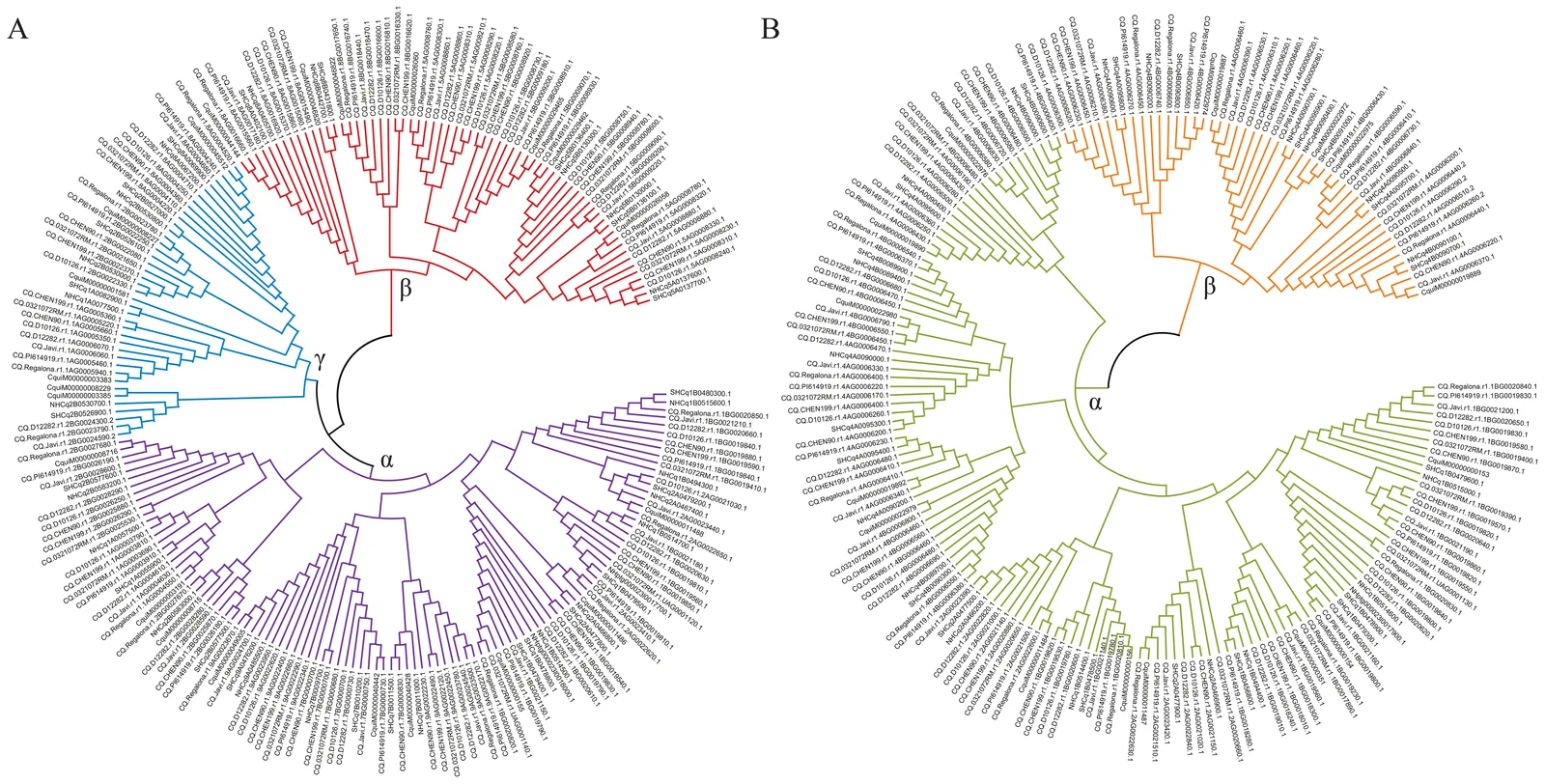
Phylogenetic analyses of the members of *CqCYP76AD*, *CqDODA* gene families. **(A)** Phylogenetic analysis of the members of *CqCYP76AD* gene family. Purple, red, and blue branches represent α, β, and γ groups of *CqCYP76AD* gene family; **(B)** Phylogenetic analysis of the members of *CqDODA* gene family. Green and orange branches represent α and β groups of *CqDODA* gene family.

Seed coat color phenotypes (white grains in highland accessions vs. dark grains in lowland accessions) were reported in previous studies (Mizuno et al., 2020) but were not independently verified or statistically compared with gene copy numbers in the current analysis. Previous studies reported that highland and lowland quinoa, though the same species, differ significantly in both genotype and phenotype, exhibiting strict environmental adaptation, for example highland varieties fail to grow at low elevations and vice versa, additionally, lowland quinoa has small, dark-colored grains, while highland quinoa produces large, white grains (Mizuno et al., 2020). Quinoa belongs to the order Caryophyllales, and its seed coat color is determined by betalains rather than anthocyanins (Sandell et al., 2024). White seed coats lack betalain accumulation, whereas black or red seed coats accumulate betalains. The core genes involved in betalain biosynthesis and accumulation in quinoa can be classified into three major categories: core biosynthetic enzyme genes, glycosyl transferase genes, and upstream regulatory transcription factors. Among them, *CqCYP76AD*, *CqDODA*, and *CqDOPA5-GT* are the key functional genes determining seed coat color (Feng et al., 2023). In betalain biosynthesis, *CqCYP76AD* and *CqDODA* encode core enzymes that convert L-DOPA to *cyclo*-DOPA and betalamic acid, respectively, and *cyclo*-DOPA is glucosylated to cDOPA 5-O-glucoside by *CqDOPA5-GT* (Chang et al., 2021; DeLoache et al., 2015). Together with the previously reported *CqCYP76AD*, *CqDODA*, and *CqDOPA5-GT* involved in betalain biosynthesis, a total of 213 *CqCYP76AD*, 184 *CqDODA*, and 20 *CqDOPA5-GT* genes were identified in the quinoa gene-based pangenome (**Supplementary Table 13****, S14, and S15**). Phylogenetic analysis revealed that the 213 *CqCYP76AD* genes clustered into three groups: CYP76AD-α, CYP76AD-β, and CYP76AD1-γ (**Figure 6A**). The CYP76AD-α group comprised 107 genes involved in the synthesis of L-DOPA and cyclo-DOPA. The CYP76AD-β group contained 41 genes functioning in tyrosine hydroxylase activity. The remaining members of the *CYP76AD* family fell into the CYP76AD1-γ branch, though their gene functions remain unknown. Based on the phylogenetic tree of the CqDODA gene family, the 184 *CqDODA* genes were divided into DODAα and DODAβ groups, with 134 genes in the DODAα group and 50 genes in the DODAβ group (**Figure 6B**).

The copy numbers of *CqCYP76AD* and *CqDODA* genes were examined across 11 quinoa genomes to assess differences between highland and lowland accessions (**Table 2**). Each genome contained at least 17 *CqCYP76AD* and 14 *CqDODA* genes. Among the *CqCYP76AD* subgroups, only CYP76AD-α showed high overall dispersion, attributable to extreme values in accessions J075 and J100. All other *CqCYP76AD* and *CqDODA* subgroups exhibited nearly identical gene numbers across the 11 genomes. Regarding *CqDOPA5-GT*, two genes were detected in most accessions, with CHEN90 (highland) and QQ74 (lowland) as exceptions. Therefore, based solely on copy number analysis, structural expansion of these betalain biosynthetic genes does not appear to be the primary driver of seed coat color divergence between highland and lowland quinoa accessions. However, it is important to emphasize that this analysis does not examine expression levels, coding sequence polymorphisms that might affect enzyme function, or regulatory variation in promoters or other cis-elements, all of which could substantially contribute to the observed color difference and warrant future investigation.

**Table 2:** Statistics of the members of *CqCYP76AD*, *CqDODA* gene families among 11 quinoa genomes.

Categories	Species	*CqCYP76AD*				*CqDODA*			*DOPA5GT*
		α	β	γ	Total	α	β	Total	Total
Highland	03-21-072RM	8	3	6	17	11	3	14	2
	CHEN-199	8	3	6	17	12	3	15	2
	CHEN-90	9	3	6	18	11	3	14	1
	D-10126	9	3	6	18	12	3	15	2
	PI-614919	9	3	6	18	13	6	19	2
	J075	14	5	5	24	13	6	19	2
	J100	12	5	5	22	13	6	19	2
Lowland	D-12282	9	3	6	18	13	5	18	2
	Javi	11	3	6	20	13	5	18	2
	QQ74	9	6	7	22	11	5	16	1
	Regalona	9	4	6	19	12	5	17	2

### Positively selected genes in quinoa pangenome

Single-copy orthologous groups were identified using OrthoFinder, yielding 335 groups shared between grape and 22 subgenomes derived from 11 quinoa genomes (Emms and Kelly, 2019). Under the branch model, four positively selected genes were detected in each quinoa individual, with grape serving as the foreground. We hypothesize that these positively selected genes may represent candidate molecular adaptations contributing to quinoa’s environmental resilience. Functional annotation indicates that these genes encode NADH-quinone oxidoreductase cyanobacterial subunit N, MT-A70, UBA-like domain-containing protein, and protein disulfide-isomerase SCO2, which are potentially involved in photosynthetic energy metabolism, RNA modification, ubiquitin-mediated protein turnover, and chloroplast protein folding, respectively (**Supplementary Table 16**). The following mechanistic interpretations are speculative and require experimental validation.

Hypothetically, in highland quinoa, positive selection might optimize these genes to cope with high-altitude stresses such as low temperature, intense ultraviolet radiation, and drought. For example, NADH-quinone oxidoreductase could potentially enhance cyclic electron transport efficiency, possibly reducing photoinhibition under harsh light. MT-A70, a component of m^6^A methyltransferase, may participate in mRNA methylation reprogramming under stress. UBA-like domain-containing protein might contribute to recognition of ubiquitinated proteins, and protein disulfide-isomerase SCO2 could facilitate chloroplast protein folding under temperature stress. Lowland quinoa may exhibit distinct selection signatures for milder environments. These hypotheses remain to be tested through functional assay.

## Discussion

### Pangenome architecture and evolutionary significance

The construction of a high-quality gene-based pangenome using 11 quinoa accessions (7 highland and 4 lowland) provides a comprehensive genomic framework for understanding the genetic diversity and evolutionary dynamics of this versatile crop. The final pangenome contained 51,298 orthologous groups, with core, softcore, dispensable, and private pan-gene sets accounting for 38.31%, 9.84%, 41.75%, and 10.1% of the total, respectively. This composition is consistent with the typical pangenome structure of crop species, where core genes form the genetic backbone essential for basic biological processes, while dispensable and private genes are candidate factors underlying phenotypic diversity and environmental adaptation. While the accumulation curve suggests a near-closed architecture within our 11-accession panel, we acknowledge that expanded sampling of wild relatives (e.g., *C. watsonii*, *C. ficifolium*) and more diverse landraces, particularly from underrepresented Andean regions, may reveal additional private or dispensable genes. Therefore, the current model reflects the saturation point within this specific panel rather than an absolute genomic boundary for the species. However, the relatively high proportion of dispensable genes (41.75%) suggests that quinoa retains substantial genetic flexibility, which may underpin its ability to adapt to diverse ecological niches, from high-altitude Andean plateaus to coastal lowlands.

The BUSCO completeness of the 11 quinoa genomes (94.5%–98.6%) confirms the high quality of gene annotations, ensuring the reliability of subsequent evolutionary and functional analyses. The core pan-genes, which are conserved across all accessions, exhibited the lowest mean Ka/Ks value (0.1172), indicating the strongest purifying selection pressure pressure—consistent with their essential roles in growth, development, and reproduction. In contrast, private pan-genes had the highest Ka/Ks value (0.1574), suggesting relatively relaxed selection and greater evolutionary plasticity, which may facilitate the generation of accession-specific adaptive traits. The enrichment of dispensable and private pan-genes in protein kinases, transcription factors, and disease resistance genes (e.g., NBS-LRR genes in private sets) further supports the notion that these non-core gene sets are key drivers of quinoa’s adaptive evolution. Specifically, the overlap in enriched gene families between dispensable and private sets implies that large gene families have undergone extensive diversification to generate genome-specific members, enabling quinoa accessions to cope with heterogeneous environmental challenges. Collectively, these findings highlight the importance of non-core pan-genes in shaping quinoa’s genetic diversity and adaptive potential, providing a valuable resource for molecular breeding and genetic improvement.

We acknowledge that the 11 quinoa genomes were annotated by different research groups using varying gene prediction pipelines, which may introduce annotation bias. While we standardized the gene sets by retaining only the longest transcript per locus and filtering transposable elements, differences in annotation parameters among the original studies likely contribute to the observed variation in total gene numbers (ranging from 42,677 to 57,061). Such heterogeneity may affect the apparent numbers of private genes, PAVs, and tandem duplications. BUSCO completeness assessment confirms overall annotation quality but does not guarantee cross-genome consistency at the individual gene level. Therefore, PAVs identified in this study should be interpreted as presence/absence at the orthologous-group level rather than definitive evidence of true gene deletion. Validation of key absences by direct mapping to raw genome assemblies represents an important direction for future work.

### Evolutionary divergence and adaptive strategies

The phylogenetic analysis of 22 A and B subgenomes (derived from 11 allotetraploid quinoa accessions) revealed distinct evolutionary trajectories between highland and lowland varieties, challenging the long-standing hypothesis that highland quinoa retains more ancient ancestral signatures. Contrary to expectations, the coastal lowland accession QQ74 exhibited closer evolutionary relationships with the outgroup grape than other accessions, indicating that it conserves the most ancient evolutionary legacy from the ancestral allotetraploid progenitors. In contrast, three highland accessions (D-10126, 03-21-072RM, and CHEN-199) showed greater divergence from the outgroup, with 03-21-072RM displaying the most recently evolved subgenomes. This pattern may be explained by the intense artificial selection and domestication pressures experienced by highland quinoa in the Andean region, which could have eroded ancient genetic signatures while promoting the emergence of adaptive traits suited to high-altitude environments (e.g., tolerance to low temperature, intense UV radiation, and drought).

The presence/absence variation (PAV) analysis further uncovered distinct adaptive strategies between highland and lowland quinoa. Highland accessions harbored more accession-specific orthologous groups (7,477 vs. 3,549 in lowland accessions) and Pfam IDs (1,112 vs. 181), with enrichment in transposon-related gene families and FAR1 DNA-binding domains. Transposon expansion enhances genomic plasticity, allowing highland quinoa to rapidly generate genetic variation in response to extreme environmental stress, while FAR1 domains optimize light signaling perception, which is critical for adaptation to high-altitude strong light conditions. Additionally, the enrichment of 4Fe-4S dicluster domains (redox sensors) and TPR/SWIM/SH3 domain protein networks suggests a coordinated mechanism to maintain cellular homeostasis under stress. In contrast, lowland quinoa prioritized metabolic efficiency and biotic defense, with specific genes enriched in ureide permeases (nitrogen assimilation), chitin recognition proteins (pathogen resistance), and Myb/SANT transcription factors (developmental regulation). This “defense-efficient, metabolically optimized” strategy is well-suited to the milder lowland environments, where resource availability and biotic stress are the primary selective pressures. It is important to emphasize that the unequal sample size (7 highland vs. 4 lowland accessions) represents a fundamental limitation of the current study. The observed difference in the number of ecotype-preferential genes (7,477 vs. 3,549 orthologous groups) is likely substantially inflated by the larger highland sample size. Without permutation or down-sampling analyses to correct for this imbalance, these PAV counts should be interpreted with caution as descriptive enrichment patterns rather than definitive evidence of altitudinal differentiation. Future pangenome studies incorporating a balanced and expanded panel of accessions are essential to rigorously quantify PAV differences between highland and lowland ecotypes.

The copy number variation of betalain biosynthetic genes (*CqCYP76AD*, *CqDODA*, and *CqDOPA5-GT*) did not correlate with seed coat color divergence between highland (white grains) and lowland (dark grains) varieties. Across the 11 genomes, we identified 213 *CqCYP76AD*, 184 *CqDODA*, and 20 *CqDOPA5-GT* genes, with each accession harboring at least 17 and 14 copies of the former two, respectively. Phylogenetic analysis revealed largely conserved copy numbers across both ecotypes, except for CYP76AD-α which showed extreme values in J075 and J100, and *CqDOPA5-GT* where CHEN90 and QQ74 contained only one copy. These findings indicate that structural genomic expansion is not the primary driver of phenotypic differentiation based on the current copy number data. Nevertheless, we acknowledge that regulatory mechanisms—such as cis-regulatory variation, transcriptional rewiring, coding sequence polymorphisms affecting enzyme activity or epigenetic modulation of betalain pathway genes—likely underlie the color divergence between altitudinal ecotypes and remain to be experimentally validated.

### Asymmetric subgenome evolution and positive selection

The asymmetric evolution of quinoa’s A and B subgenomes, manifested in biased gene retention and tandem duplication, provides critical insights into the consequences of polyploidization. Subgenome B retained more single-copy genes than subgenome A, while two-copy retained genes (dosage-sensitive genes) were twice as abundant as single-copy genes across all accessions. This pattern aligns with previous reports of subgenome dominance in polyploids, where one subgenome maintains more ancestral gene content while the other undergoes greater divergence. The enrichment of two-copy retained genes in disease resistance-related domains (e.g., NB-ARC, Rx N-terminal) suggests that dosage sensitivity plays a key role in quinoa’s biotic stress tolerance, while single-copy genes (enriched in protein kinases and transcription factors) may regulate core developmental processes. Additionally, tandem duplicated genes were more abundant in subgenome B than A, with enrichment in abiotic stress, related families (e.g., salt stress response, peroxidase), indicating that tandem duplication contributes to quinoa’s adaptation to harsh environments. Notably, dosage-sensitive genes (from polyploidization) and tandemly duplicated genes (from tandem duplication) target distinct stress responses (biotic vs. abiotic), highlighting the complementary roles of these two evolutionary mechanisms in enhancing quinoa’s environmental adaptability.

The identification of four positively selected genes represents a preliminary inference of candidate loci underlying altitudinal adaptation. While PAML analysis supports elevated dN/dS ratios in these genes, the following functional interpretations are speculative. These genes are annotated as being involved in photosynthetic energy metabolism, RNA modification, protein homeostasis, and chloroplast protein folding—processes that may be relevant to environmental stress responses. We hypothesize that divergent amino acid variations between highland and lowland populations could potentially contribute to ecological divergence, but direct experimental evidence linking these specific substitutions to phenotypic differences in photosynthesis, UV tolerance, or stress resistance is currently lacking. Future studies using transgenic or gene-editing approaches are required to validate the functional roles of these candidate genes before they can be considered for molecular breeding applications.

In conclusion, this study provides a comprehensive analysis of the quinoa gene-based pangenome, revealing key insights into subgenome evolution, highland-lowland divergence, and adaptive strategies. The findings highlight the importance of non-core pan-genes, asymmetric subgenome evolution, and positively selected genes in shaping quinoa’s genetic diversity and environmental adaptability. Future research should focus on functional validation of candidate adaptive genes, exploration of epigenetic regulatory mechanisms, and integration of pan-genomic data into molecular breeding programs to improve quinoa’s yield, quality, and stress tolerance.

## Materials and methods

### Data retrieval

The genomic data of 11 quinoa accessions, including assembled genome sequences, coding sequences, predicted protein sequences, and gene annotation files, were downloaded from quinoaDB (https://quinoadb.org) (Jarvis et al., 2017). For genes with alternative splicing evidence, only the longest transcript was retained as the representative sequence for each gene. Transposable elements (TEs) were filtered from the protein sequences using TEsorter v1.4.6 with the parameters “-st prot -p 160 -cov 30 -eval 1e-5 -dp2” (Zhang et al., 2022), followed by an additional filtering step based on the keyword “transposon” in the InterProScan v5.52-86.0 annotation (Jones et al., 2014). After curation, a total of 504,240 high-quality protein-coding genes from the 11 quinoa genomes were retained for downstream analyses. The completeness of each genome was assessed using Benchmarking Universal Single-Copy Orthologs (BUSCO) version 5.4.7 (Berkeley et al., 2021), and only accessions with BUSCO completeness scores > 94% were retained for subsequent analyses.

### Construction of gene-based pangenome

Orthologous groups were identified among the 11 quinoa genomes using OrthoFinder v2.5.4 with default parameters (Emms and Kelly, 2019). The resulting output files, including orthogroups and unassigned genes files, were merged to generate the final dataset of quinoa orthologous groups for downstream analyses. Based on their presence across genomes, these groups were classified into four categories: core genes (present in all 11 genomes), softcore genes (present in10–11 genomes, i.e., ≥90.9%), dispensable genes (present in 2–9 genomes), and private genes (present in only one genome). We note that with only 11 genomes, a >99% threshold is mathematically equivalent to 100%; therefore, we adopted a ≥90.9% (10/11) threshold for softcore to ensure a meaningful distinction from the core set. Pangenome and core-genome accumulation curves were generated by iterative random sampling of genome combinations (1,000 permutations per accession number). The pan-genome size was modeled using a power-law function (y = ax^b + c) and an exponential decay function (y = a·e^(bx) + c), with the best-fit model selected based on the Akaike information criterion (AIC). Ninety-five percent confidence intervals were derived from 1,000 bootstrap resamplings of genome addition order.

### Phylogenetic analysis of the subgenomes of quinoa accessions

All quinoa protein-coding genes were assigned to either the A or B subgenome based on their chromosomal locations, and genes with unassigned positions were excluded. This resulted in a total of 22 subgenomic datasets: 11 A subgenomes and 11 B subgenomes. *V. vinifera* was designated as the outgroup species. A total of 5,318 single-copy orthologs shared among the 22 quinoa subgenomes and *V. vinifera* were identified and used for phylogenetic analysis. Multiple sequence alignments were performed using MAFFT v7.520 (Katoh et al., 2019), and conserved blocks were extracted from the aligned sequences using Gblocks 0.91b with the parameters “-b4 = 5 -b5=h -t=p -e=.2” (Castresana, 2000). The conserved sequences from each sample were then concatenated into a single supermatrix using SeqKit v0.3.1.1 with the parameter “-w” (Shen et al., 2016). Phylogenetic trees were reconstructed using IQ-TREE v2.3.1 with the parameters “-m MFP -bb 1000 -bnni -T 30” (Minh et al., 2020).

### Retrieval of orthologous gene pairs across subgenomes

Orthologous gene pairs were identified by performing all-against-all protein sequence comparisons among the 22 quinoa subgenomes (11 A and 11 B) and *V. vinifera* using DIAMOND BLASTP with an e-value threshold of 1e-5 (Buchfink et al., 2021). The top-scoring reciprocal best hits were retained as candidate orthologous pairs for downstream syntenic analysis. Syntenic orthologous gene pairs were subsequently identified using MCScanX (version 11.13.2012) with the following parameters: e-value ≤ 1e-20, maximum gap between genes (u) = 1, and minimum number of anchor points (s) = 15 (Wang et al., 2024). These syntenic orthologous gene pairs were then used to investigate the asymmetric evolution of subgenomes across the quinoa genomes.

### Analysis of tandemly duplicated genes

Paralogous gene pairs within each quinoa genome were identified by performing all-against-all protein sequence comparisons using DIAMOND BLASTP with an e-value threshold of 1e-5 (Buchfink et al., 2021). Tandemly duplicated genes were defined as adjacent paralogous genes located within 100 kb on the same chromosome, with ≥70% amino acid sequence identity, ≥70% alignment coverage, and no more than 10 intervening non-paralogous genes.

### Analysis of pan-gene sets for *K*a, *K*s, and *K*a/*K*s

KaKs_Calculator 2.0 was employed to analyze rates of non-synonymous (*K*a) and synonymous (*K*s) substitutions, as well as the *K*a/*K*s ratio, for the orthologous gene pairs between each quinoa genome and *V. vinifera* (Wang et al., 2010). This analysis independently generated 11 output files for *K*a, *K*s, and *K*a/*K*s, respectively. All *K*a, *K*s, and *K*a/*K*s value files were merged into separate consolidated datasets. Based on the gene lists of the four pan-gene sets (core, softcore, dispensable, and private), the merged *K*a, *K*s, and *K*a/*K*s datasets were then categorized accordingly. These categorized values were subsequently used to infer evolutionary patterns and selection constraints across different pan-gene sets.

### Pfam and GO enrichment analysis

Pfam IDs and GO terms of quinoa genes were extracted from InterProScan output files. Pfam enrichment analyses were conducted using in-house scripts. For each target gene set, a 2×2 contingency table was constructed as follows: (1) Foreground: the number of genes annotated with each Pfam ID within the target gene set, and the total number of genes in that set; (2) Background: the number of genes annotated with the same Pfam ID across the entire pangenome, and the total number of genes in the pangenome. Fisher’s exact test was then performed to statistically assess the enrichment of each Pfam ID, with an adjusted P-value < 0.05 as the significance threshold. Gene Ontology enrichment analyses were performed using the clusterProfiler v4.2 R package, with the same significance threshold (adjusted P-value < 0.05) (Wu et al., 2021).

### PAV analysis of genes and gene families

Analyses of presence/absence variation (PAV) classified quinoa pan-genes into two gene sets corresponding to highland and lowland accessions based on their distribution across orthologous groups. To elucidate genetic variation between these ecotypes, orthologous groups were categorized according to the taxonomic affiliation of quinoa accessions, with a specific focus on PAV patterns of genes and gene families distinguishing highland from lowland quinoa. Subsequent downstream analyses were primarily concentrated on orthologous groups specific to either the highland or lowland group.

### Identification of seed coat color related genes

Based on previously reported betalain biosynthesis genes, protein sequences of *CYP76AD*, *DODA*, and *DOPA5-GT* from representative betalain-producing species, including *Amaranthus cruentus*, *Beta vulgaris*, *Carnegiea gigantea*, *Mirabilis jalapa*, and *Bougainvillea spectabilis*, were retrieved from the NCBI database. Retrieved sequences were curated to remove redundancies and used as queries in a DIAMOND BLASTp search against the 11 quinoa genomes, with a sequence identity threshold of ≥50% (Buchfink et al., 2021). Candidate genes were subsequently validated by conserved domain verification using InterProScan v5.52-86.0 package (Jones et al., 2014). Only candidates containing canonical functional domains were retained as the final set of betalain biosynthesis genes (*CqCYP76AD*, *CqDODA*, and *CqDOPA5-GT*). Phylogenetic trees of the quinoa betalain gene families were constructed following the same procedures used for the subgenome phylogenetic analysis.

### Analysis of positively selected genes

A total of 5,318 single-copy orthologs shared among the 22 quinoa subgenomes (11 A and 11 B) and *V. vinifera* were identified and used for phylogenetic analysis. For positive-selection analysis, we applied additional quality filters (alignment length >300 bp, removal of excessive gaps) to retain 335 high-confidence orthologous groups suitable for PAML analysis. These 335 orthologs were used for the analysis of positively selected genes (PSGs) in the quinoa clade. Multiple sequence alignments of coding sequences were performed using MAFFT v7.520 (Rozewicki et al., 2019) and PAML v4.4b (Yang, 2007) was employed to estimate dN/dS ratios.

i) Branch model analysis. The dN/dS ratios were initially estimated using branch models (model = 2, NSsites = 0; Zhao et al., 2010) with the following parameters: CodonFreq = 2, kappa = 2.5, and initial omega = 0.2. Three hypotheses were tested: (i) H0 (null hypothesis), assuming an identical dN/dS ratio across all branches; (ii) H1 (alternative hypothesis), assuming a distinct dN/dS ratio for the target quinoa branch while all other branches share an identical ratio; and (iii) H2 (unconstrained hypothesis), assuming different dN/dS ratios for all branches. A likelihood-ratio test (LRT) was performed to identify candidate genes satisfying two criteria: (1) the likelihood value of H1 was significantly greater than that of H0 (adjusted LRT P-value < 0.01), and (2) the likelihood value of H2 was not significantly greater than that of H1.
ii) Branch-site model analysis. To detect positively selected sites in the target quinoa groups, branch-site models (model = 2, NSsites = 2) were employed. The null hypothesis was specified with fix_omega = 1 and omega = 1, while the alternative hypothesis used fix_omega = 0 and omega = 1.5 with the inferred phylogenetic tree. Genes with positively selected sites were identified using an FDR-corrected LRT with an adjusted P-value cutoff of < 0.01.

## Data Availability

The original contributions presented in the study are included in the article/**Supplementary Material**. Further inquiries can be directed to the corresponding author.
